# Supplementary material for: Brown marmorated stink bug overwintering aggregations are not regulated through vibrational signals during autumn dispersal
Source: R Soc Open Sci. 2020 Nov 18;7(11):201371. doi: 10.1098/rsos.201371 (PMC7735358; doi:10.1098/rsos.201371)
Supplement: Supplementary information [file rsos201371supp2.pdf]

Supplementary information

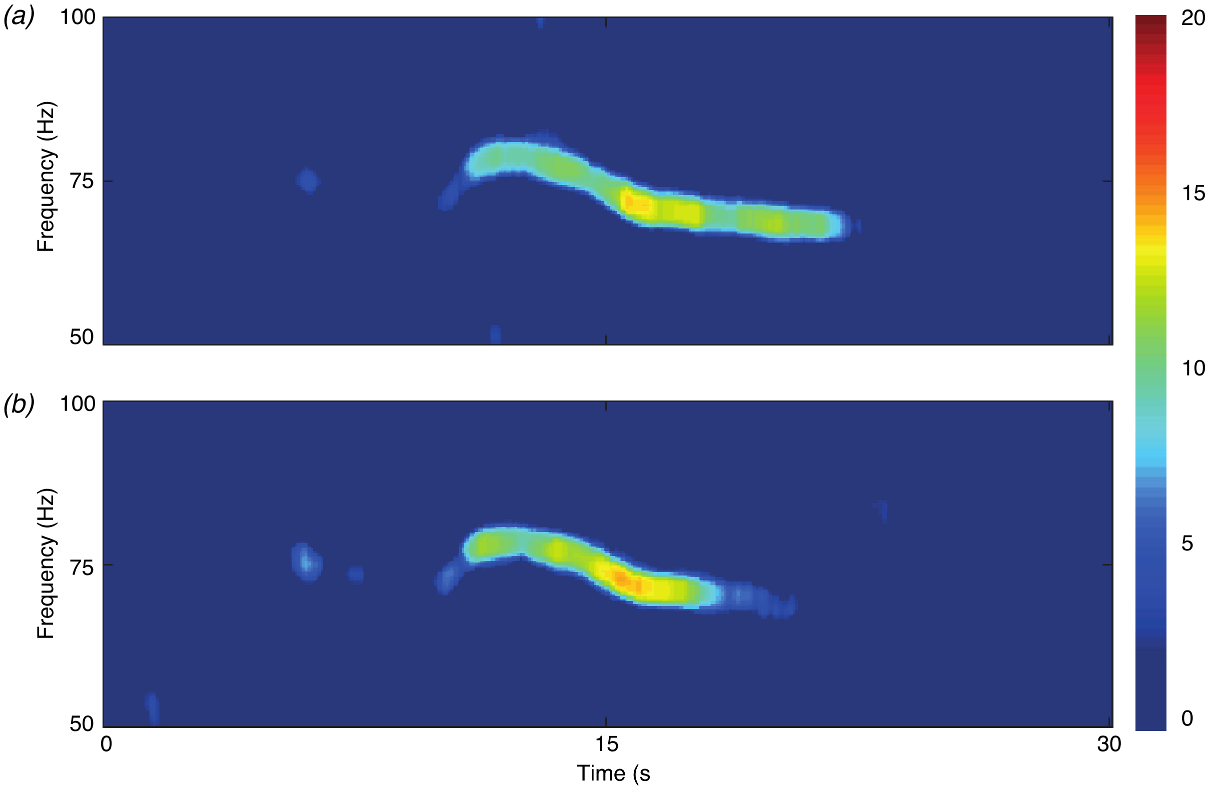

Figure S1. Spectrograms of the vibrational signal of a male *H. halys* simultaneously recorded with two different types of sensors inside the same shelter. Top: Piezoelectric microphone; Bottom: accelerometer. The signals are not identical, but are equivalent for acoustic-detection purposes. Colourbar in dB.

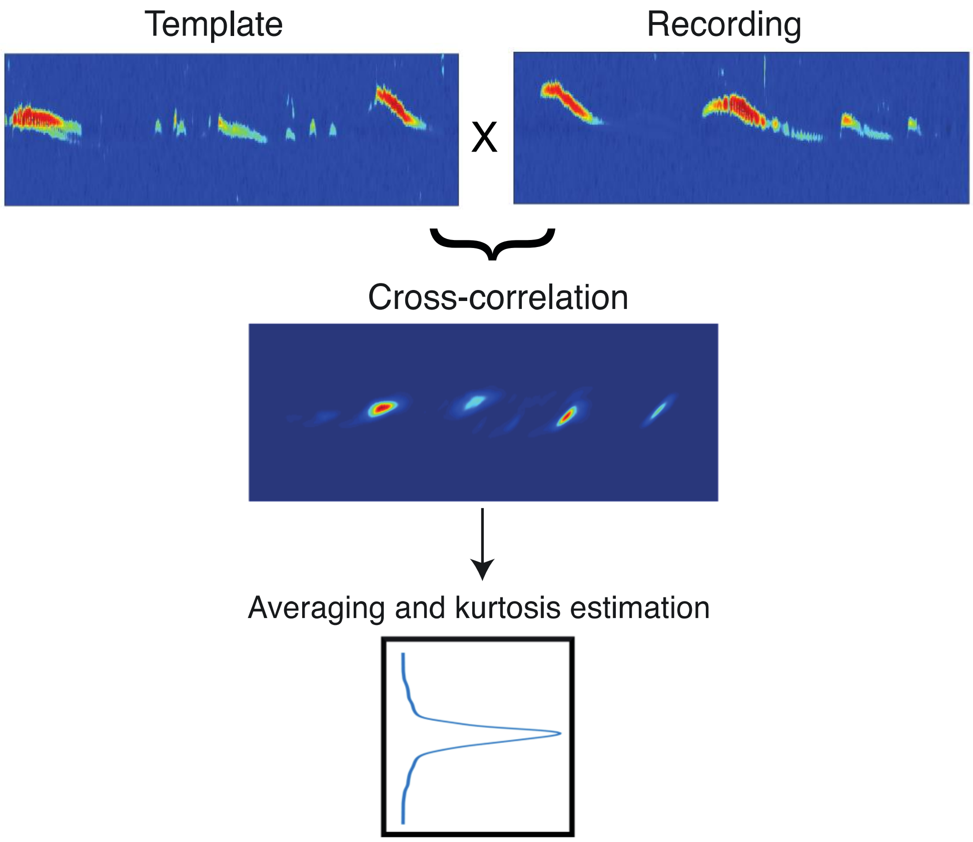

Figure S2. Pattern matching method used for the automatic detection of *H. halys* vibrations. The method estimates the 2D cross-correlation between the spectrogram of a signal template and 1-min sections of the dataset. Then, the kurtosis of the marginal distribution of values in the spectral domain is used as detection criterion for the presence of *H. halys* vibrations, which generate leptokurtic distributions. Both signals (template and recording) are 1 minute long and have the same spectral range (30 to 100 Hz).

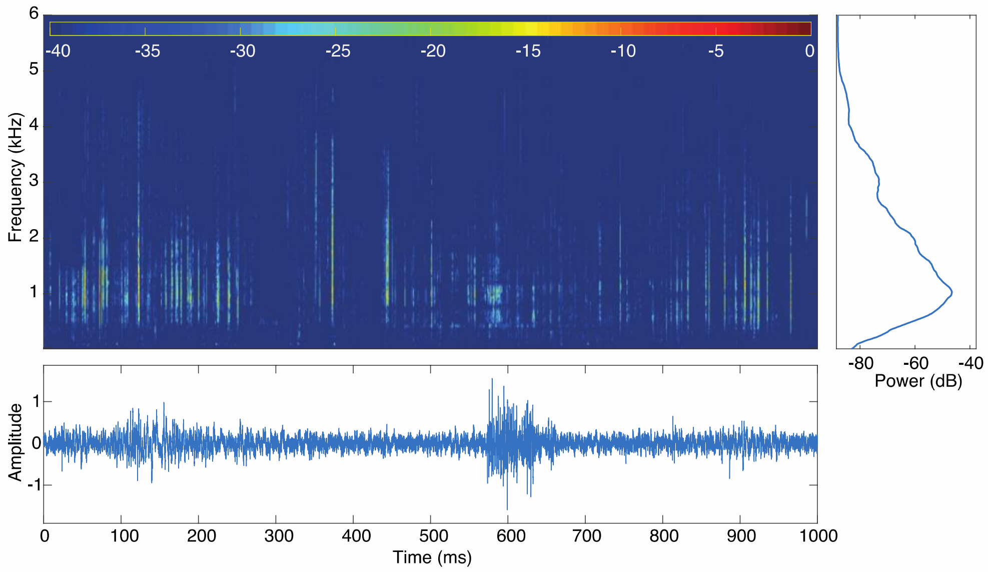

Figure S3. Characterization of *H. halys* movement inside the wooden shelters. Mechanical vibrations of *H. halys* can be detected by analysing variations in the power spectral distribution between 500 Hz - 5 kHz. Power intensity is directly correlated with the amount of movement.
